# Loss of ZBED6 Protects Against Sepsis‐Induced Muscle Atrophy by Upregulating DOCK3‐Mediated RAC1/PI3K/AKT Signaling Pathway in Pigs

**DOI:** 10.1002/advs.202302298

**Published:** 2023-08-07

**Authors:** Huan Liu, Dengke Pan, Pu Li, Dandan Wang, Bo Xia, Ruixin Zhang, Junfeng Lu, Xiangyang Xing, Jiaxiang Du, Xiao Zhang, Long Jin, Lin Jiang, Linong Yao, Mingzhou Li, Jiangwei Wu

**Affiliations:** ^1^ Key Laboratory of Animal Genetics Breeding and Reproduction of Shaanxi Province College of Animal Science and Technology Northwest A&F University Yangling Shaanxi 712100 China; ^2^ Clinical Immunology Translational Medicine Key Laboratory of Sichuan Province Sichuan Academy of Medical Sciences & Sichuan Provincial People's Hospital Chengdu Sichuan 610072 China; ^3^ Department of Critical Care Medicine the Second Affiliated Hospital of Air Force Medical University No.569, Xinsi Road Xi'an Shaanxi 710038 China; ^4^ Laboratory of Animal (Poultry) Genetics Breeding and Reproduction Ministry of Agriculture Institute of Animal Sciences Chinese Academy of Agricultural Sciences (CAAS) Beijing 100193 China; ^5^ Chengdu Clonorgan Biotechnology Co. LTD Chengdu Sichuan 610041 China; ^6^ Institute of Animal Genetics and Breeding College of Animal Science and Technology Sichuan Agricultural University Chengdu Sichuan 611130 China

**Keywords:** DOCK3, muscle atrophy, sepsis, ZBED6

## Abstract

Sepsis‐induced muscle atrophy often increases morbidity and mortality in intensive care unit (ICU) patients, yet neither therapeutic target nor optimal animal model is available for this disease. Here, by modifying the surgical strategy of cecal ligation and puncture (CLP), a novel sepsis pig model is created that for the first time recapitulates the whole course of sepsis in humans. With this model and sepsis patients, increased levels of the transcription factor zinc finger BED‐type containing 6 (ZBED6) in skeletal muscle are shown. Protection against sepsis‐induced muscle wasting in ZBED6‐deficient pigs is further demonstrated. Mechanistically, integrated analysis of RNA‐seq and ChIP‐seq reveals dedicator of cytokinesis 3 (DOCK3) as the direct target of ZBED6. In septic ZBED6‐deficient pigs, DOCK3 expression is increased in skeletal muscle and myocytes, activating the RAC1/PI3K/AKT pathway and protecting against sepsis‐induced muscle wasting. Conversely, opposite gene expression patterns and exacerbated muscle wasting are observed in septic ZBED6‐overexpressing myotubes. Notably, sepsis patients show increased ZBED6 expression along with reduced DOCK3 and downregulated RAC1/PI3K/AKT pathway. These findings suggest that ZBED6 is a potential therapeutic target for sepsis‐induced muscle atrophy, and the established sepsis pig model is a valuable tool for understanding sepsis pathogenesis and developing its therapeutics.

## Graphical abstract

Sepsis‐induced muscle atrophy often increases morbidity and mortality in intensive care unit (ICU) patients, yet neither a therapeutic target nor an optimal animal model is available for this disease. Here, a novel sepsis pig model is created which for the first time, recapitulates the whole course of sepsis in humans. Targeting zinc finger BED‐type containing 6 (ZBED6) for sepsis‐induced muscle atrophy evidence from humans and pigs.

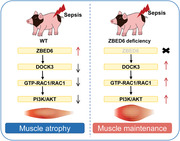

## 1 Introduction

Up to 90% of severe sepsis patients have muscle atrophy that hampers weaning from ventilatory support, delays rehabilitation, and is associated with an increased risk of death.^[^ ^1^ ^]^ Although various contributing factors such as inflammation,^[^ ^2^ ^]^ oxidative stress^[^ ^3^ ^]^ and mitochondrial dysfunction,^[^ ^4^ ^]^ and protein metabolism imbalance^[^ ^5^ ^]^ have been identified, the molecular regulators involved in modulating muscle atrophy remain poorly understood. Several animal models, such as pig,^[^ ^6^ ^]^ rat,^[^ ^7^ ^]^ and mouse,^[^ ^8^ ^]^ have played a crucial role in advancing our knowledge of sepsis‐induced muscle atrophy. However, it is essential to recognize the inherent limitations of these animal models and their inability to fully mimic the intricate complexities of the human system.^[^ ^9^ ^]^ For example, rodents have limited translational value due to their heightened resistance to pathogens and sepsis compared to humans.^[^ ^9^, ^10^ ^]^ Accumulating evidence support that pigs are appropriate models for studying sepsis due to their immune system, anatomy, genome, and physiology closely resembles that of humans.^[^ ^11^ ^]^

However, the current sepsis pig model created by conventional cecal ligation and puncture (CLP) has limited applicability due to high acute mortality rates within 48–96 h of the procedure.^[^ ^12^ ^]^ Therefore, it cannot be used to study the long‐term host response to sepsis, such as sepsis‐induced muscle atrophy. Consequently, modified pig models are urgently needed to advance our understanding and treatment of sepsis‐induced muscle atrophy and other sepsis‐related conditions.

Zinc finger BED‐type containing 6 (ZBED6) is a highly conserved transcription factor that was identified by comparing the genomes of wild and domesticated pigs.^[^ ^13^ ^]^ Structurally, ZBED6 contains two DNA‐binding BED domains which remain identical across 25 placental mammals. Its crucial role in regulating gene programs that control various cellular processes and cell states has attracted significant interest in health and disease.^[^ ^13^, ^14^ ^]^ We and others have shown that ZBED6 deficiency promotes postnatal skeletal muscle development in pigs and mice both,^[^ ^13^, ^14^, ^15^ ^]^ suggesting that ZBED6 serves as a negative regulator of muscle growth under physiological conditions. Furthermore, ZBED6 has been implicated in the development of various diseases, such as colorectal cancer,^[^ ^16^ ^]^ colitis,^[^ ^17^ ^]^ and diabetes.^[^ ^14^, ^18^ ^]^ The role of ZBED6 in the regulation of these pathologies prompted us to consider whether ZBED6 plays a significant role in sepsis‐induced muscle atrophy, and if so, how does it regulate this pathological process?

As a transcription factor, ZBED6 regulates the transcription of a series of downstream target genes by recognizing and binding a specific DNA sequence (GCTCGC).^[^ ^14b^ ^]^ Insulin‐like growth factor 2 (IGF2) was the first identified target of ZBED6 during normal muscle growth in pigs and mice.^[^ ^13^ ^]^ We previously showed that ZBED6 regulates multiple new target genes such as cyclin‐dependent kinase inhibitor 1A (CDKN1A) in skeletal muscle other than IGF2. Dedicator of cytokinesis 3 (DOCK3) is a regulator of muscle atrophy.^[^ ^19^ ^]^ Genetic deletion of Dock3 in mice results in muscle atrophy and DOCK3‐deficient myoblasts are defective for myogenic differentiation.^[^ ^19b^ ^]^ Individuals with loss‐of‐function DOCK3 variants develop ataxia and developmental delay and have low muscle tone from birth.^[^ ^20^ ^]^ DOCK3 rs77031559 G is associated with muscle fiber size and strength in elite athletes.^[^ ^21^ ^]^ These findings highlight the essential role of DOCK3 in the regulation of muscle growth and atrophy. Intriguingly, our preliminary sequence analysis reveals putative ZBED6 binding site in the promoter region of porcine DOCK3, suggesting a potential link between ZBED6 and DOCK3 that might be crucial for muscle atrophy.

Thus, to explore whether ZBED6 plays a key role in the regulation of sepsis‐induced muscle atrophy with a suitable model, we developed a modified CLP pig model to mimic the whole course of sepsis in the presence or absence of ZBED6 (ZBED6‐deficient pigs and WT controls). By this model, we show that deficiency of ZBED6 prevents sepsis‐induced muscle atrophy. Through integrated RNA‐seq and ChIP‐seq analysis, we identified DOCK3 as the direct negative target of ZBED6 in skeletal muscle of pigs under septic conditions. ZBED6 deficiency increased DOCK3 expression, leading to hyperactivation of its downstream RAC1/PI3K/AKT signaling cascade, thus preventing sepsis‐induced muscle atrophy. Our findings suggest that ZBED6 is a promising therapeutic target for sepsis‐induced muscle atrophy, with potential clinical applications for the treatment of muscle loss in sepsis. Furthermore, our innovative sepsis pig model serves as a powerful tool for facilitating mechanistic exploration of sepsis‐induced muscle wasting and other related multi‐organ injuries, as well as for developing and testing potential therapeutics, thus providing an idea experimental model for pre‐clinical sepsis studies.

## 2 Results

### 2.1 Sepsis‐Induced Muscle Loss is Associated with the Upregulation of ZBED6 in Sepsis Patients

**Figure 1:**
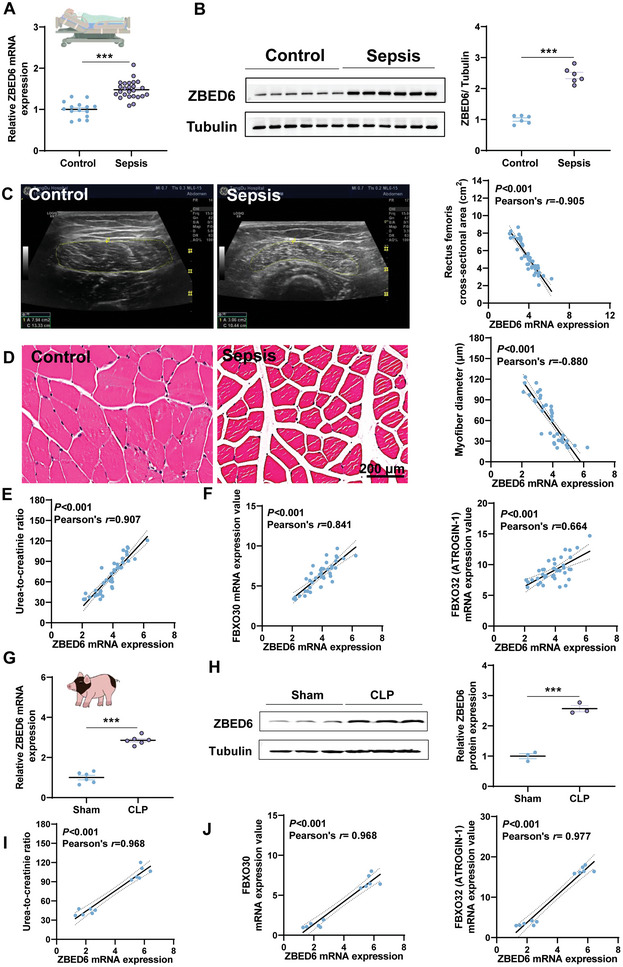
Upregulation of ZBED6 in sepsis‐induced muscle loss for humans and pigs. A) mRNA expression of ZBED6 in muscle biopsies of 25 sepsis patients with muscle loss and 15 orthopedic controls. B) Immunoblot and densitometric analysis of ZBED6 in rectus femoris muscle tissues of sepsis patients and controls (*n* = 6). Representative images are shown. C) The rectus femoris cross‐sectional area (RF‐CSA) was measured using ultrasound imaging in sepsis patients and orthopedic controls. The area was outlined by a dotted line. Scatterplots showing correlations of ZBED6 transcript with RF‐CSA in sepsis patients and orthopedic controls. D) H&E staining of histological cross sections in rectus femoris muscle tissues of sepsis patients and controls. Representative images are shown. Scale bar = 200 µm. The mean minimum Feret diameter of muscle fibers from sepsis patients and controls were correlated with ZBED6 transcript expression. E) Scatterplots showing correlations of ZBED6 transcript with urea‐to‐creatinine ratio in sepsis patients and controls. F) Scatterplots showing correlations of ZBED6 transcript with the transcripts of FBXO30 and FBXO32 in the rectus femoris muscle tissues of sepsis patients and those of controls. G) Bama pigs were subjected to sham or modified CLP surgery (*n* = 6). mRNA expression of ZBED6 in skeletal muscles of sham and modified CLP pigs 14 days post‐surgery. (H) Immunoblot and densitometric analysis of ZBED6 in skeletal muscle from sham and modified CLP pigs (*n* = 3). I) Scatterplots showing correlations of ZBED6 transcript with urea‐to‐creatinine ratio in the sham and modified CLP pigs. J) Scatterplots showing correlations of ZBED6 transcript with *FBXO32* and *FBXO30* in skeletal muscle of sham and modified CLP pigs. Data are expressed as mean ± SEM; *p < 0.05, **p < 0.01, ***p < 0.001.

To determine whether ZBED6 is implicated in sepsis‐induced muscle atrophy, we collected muscle biopsies and plasma samples from 25 sepsis patients with muscle loss (patients lost more than 10% of the cross‐sectional area (CSA) of the rectus femoris (RF‐CSA) were recruited) and 15 orthopedic controls. Patient characteristics were shown in Table S1, Supporting Information. High mRNA and protein levels of ZBED6 were shown in muscle tissues of the sepsis patients (**Figure** **1**A,B). We further performed quantitative muscle ultrasound in these individuals and found negative correlation between RF‐CSA, a marker for muscle atrophy, and the mRNA expression of ZBED6 (Figure 1C). Histological evaluation of muscle fibers by H&E staining revealed a negative correlation between ZBED6 transcript expression and the mean minimum feret diameter, a parameter of muscle atrophy (Figure 1D). Furthermore, we found that mRNA expression levels of ZBED6 were positively correlated with urea‐to‐creatinine ratio, a biochemical marker for muscle catabolism (Figure 1E), and with those of the well‐recognized muscle atrophy marker genes f‐box protein 32 (*FBXO32*) and f‐box protein 30 (*FBXO30*) (Figure 1F). Together, these results suggest that the upregulation of ZBED6 may be a crucial factor contributing to sepsis‐induced muscle atrophy. Further studies, including validation in animal models, are necessary to verify this association and provide additional insight into the underlying mechanisms.

### 2.2 Creation of a Modified Sepsis Pig Model that Allows for Long‐Term Investigation of Sepsis‐Induced Muscle Atrophy

To explore the role of ZEBD6 in sepsis‐induced muscle atrophy, a suitable sepsis animal model is required. Pigs are ideal models for sepsis simulation given their similarities to humans in immune system, anatomy, genome, and physiology. However, the high acute mortality rate in the current sepsis pig model created by CLP limits its use. To overcome this limitation, we developed a modified pig CLP model by changing the surgical procedure to cecal incision and interrupted sutures, and providing supportive therapeutic interventions such as rapid volume resuscitation, analgesia, and anti‐inflammatory measures (Figure S1A, Supporting Information). Compared with the conventional method which exhibited 100% of death in pigs within 4 days of the surgery and an average survival time of 2.33 ± 0.42 days (Figure S1B, Supporting Information), the modification greatly reduced the mortality rate of pigs (Figure S1B, Supporting Information) and better represented the progression and features of human sepsis, showing persistent fever (Figure S1C, Supporting Information), sustained weight loss (Figure S1D, Supporting Information), immunosuppression (Figure S1E, Supporting Information), systemic inflammation (Figure S1F, Supporting Information), multiorgan injury (liver, Figure S2A–D, Supporting Information; kidney, Figure S2E–J, Supporting Information; lung injury, Figure S2K, Supporting Information) and muscle atrophy (Figure S2L–P, Supporting Information). Moreover, the novel pig CLP model satisfies the Minimum Quality Threshold in Pre‐Clinical Sepsis Studies (MQTiPSS) guidelines, making it a suitable experimental model for preclinical sepsis studies. Together, the modified pig CLP model offers more flexibility in investigating sepsis development and allows for mechanistic exploration of sepsis‐induced muscle atrophy.

### 2.3 Sepsis‐Induced Muscle Loss is Associated with Increased ZBED6 Expression in Septic Pigs

With the modified CLP model, we showed that septic pigs had significantly higher levels of ZBED6 mRNA and protein compared to sham controls (Figure 1G,H). Furthermore, we observed positive correlations between *ZBED6* mRNA expression and the urea‐to‐creatinine ratio (Figure 1I) and the expression of muscle atrophy marker genes (Figure 1J) in septic pigs. Together, these results suggest a potential association between sepsis‐induced muscle loss and increased ZBED6 expression in septic pigs.

### 2.4 ZBED6 Deficiency Protects Against Sepsis‐Induced Muscle Atrophy in Pigs

**Figure 2:**
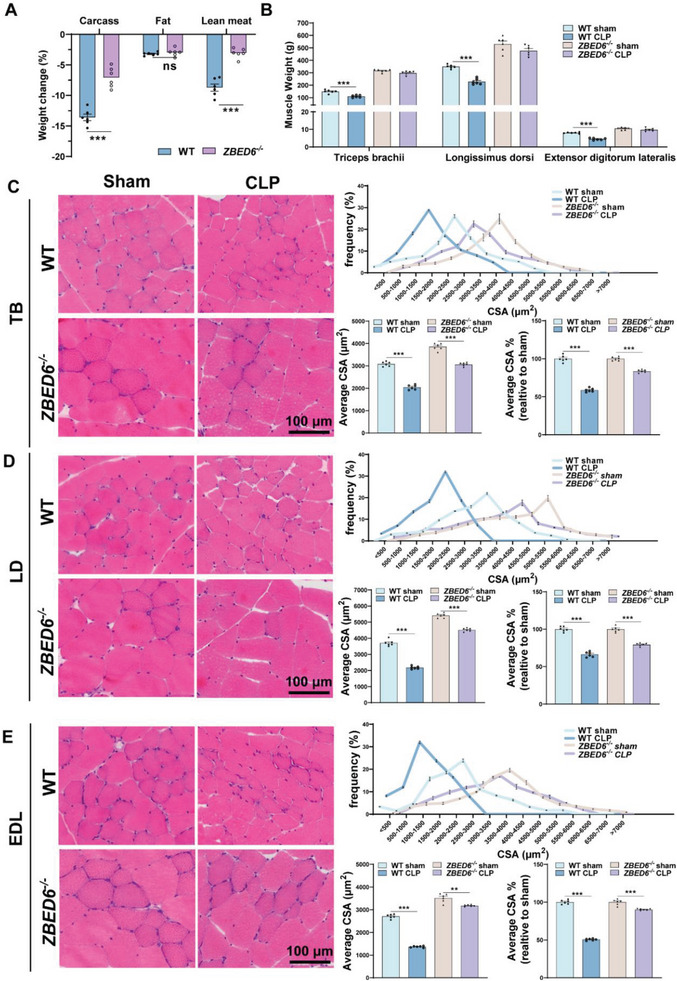
ZBED6 deficiency protects against sepsis‐induced muscle atrophy in pigs. ZBED6‐deficient and WT pigs were subjected to modified CLP or sham surgery. After 14 days, the pigs were sacrificed. A) Loss of total body weight, fat mass, and lean mass expressed as percent‐wise change compared with the respective sham group. B) Muscle weight of Triceps brachii (TB), Longissimus dorsi (LD), and Extensor digitorum lateralis (EDL). C–E) Presentative images of H&E staining of histological cross sections from TIP (C), LD (D), and EDL (E). Scale bar = 100 µm. (Right top) Frequency of distribution for myofiber CSA (µm^2^) of TB (C), LD (D), and EDL E) muscles (*n*  =  6 per group). (Right bottom) Quantification of average CSA and average CSA% (expressed as percent‐wise change compared with their respective sham group) from H&E staining. One hundred myofibers were measured for each pig. Data are expressed as mean ± SEM; *p < 0.05, **p < 0.01, ***p < 0.001.

To further determine the role of ZBED6 in sepsis‐induced muscle atrophy, we generated septic ZBED6‐deficient pigs (*ZBED6^−/−^*) based on our previous *ZBED6^−/−^* pigs.^[^ ^14a^ ^]^ Two weeks after surgery, sepsis caused smaller weight reductions of carcass (CW, 7.05% vs 13.60%), carcass fat (2.96% vs 3.20%), and carcass lean meat (CLW, 3.08% vs 8.71%) (**Figure** **2**A) in *ZBED6^−/−^* pigs than in WT pigs when compared to their respective controls. A similar average daily food intake was observed in two groups of pigs (Figure S3A,B, Supporting Information), suggesting that prevention of sepsis‐induced muscle atrophy in ZBED6‐deficient pigs was not attributed to increased energy intake. ZBED6 depletion attenuated 48.16% loss in CW (*P* < 0.001, Figure 2A), which was mainly due to the prevention of loss in CLW (*P* < 0.001, Figure 2A). Having observed the loss in systemic muscle weights, we then measured the depot‐specific loss from triceps brachii (TB, forelimb), longissimus dorsi (LD, trunk), and extensor digitorum lateralis muscles (EDL, hindlimb), showing consistent resistance to sepsis‐induced loss upon ZBEB6 deficiency (Figure 2B). Histologically, septic WT pigs showed smaller average CSA (Figure 2C–E) and higher percentage of myofibers with smaller CSA compared with WT sham controls (Figure 2C–E). Nonetheless, ZBED6 deficiency greatly attenuated sepsis‐induced reduction in myofiber CSA, without obviously affecting the relative frequency distribution (Figure 2C–E).

**Figure 3:**
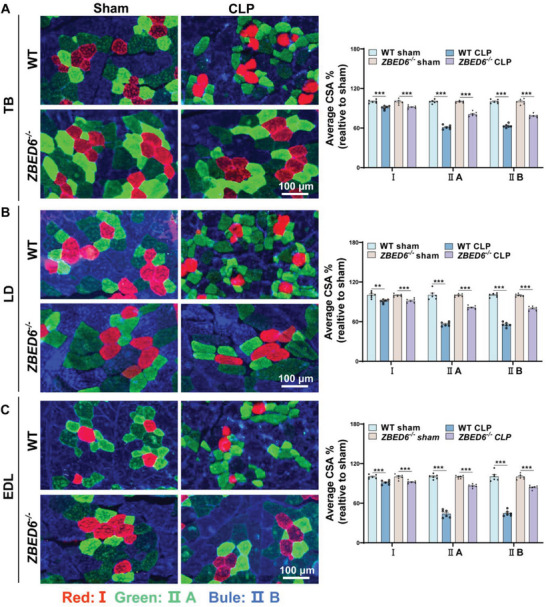
ZBED6 deficiency attenuates type II myofiber atrophy in septic pigs. A–C) (Left) Representative images of fiber‐type staining of the TB (A), LD (B), and EDL (C) muscles showing myosin heavy chain type I fibers (red), IIa (green), and IIb (bule); scale bars represent 100 µm. (Right) CSA analysis by fiber type. Average CSA% expressed as percent‐wise change compared with the respective sham group. *n* =  6 per group. Data are expressed as mean ± SEM; *p < 0.05, **p < 0.01, ***p < 0.001.

Accumulating clinical studies have shown that patients with sepsis experience a preferential reduction in type II muscle fibers.^[^ ^22^ ^]^ To provide a more comprehensive assessment of atrophy and potential fiber‐type variations in the sepsis condition, we conducted an in‐depth evaluation of myofiber‐specific changes and revealed a mild reduction in CSA of type I fibers (<10%) and a severe loss in type II fibers (37%−56%) in septic WT pigs (**Figure** **3**A–C). In contrast, small reductions in CSA of type II fibers were observed in ZEBD6‐deficient pigs (14%−22%) in the context of sepsis (Figure 3A–C). Together, these results suggest that depletion of ZBED6 protects against sepsis‐induced muscle atrophy.

### 2.5 Loss of ZBED6 Rescues an Imbalance in Protein Metabolism During Sepsis‐Induced Muscle Atrophy in Pigs

**Figure 4:**
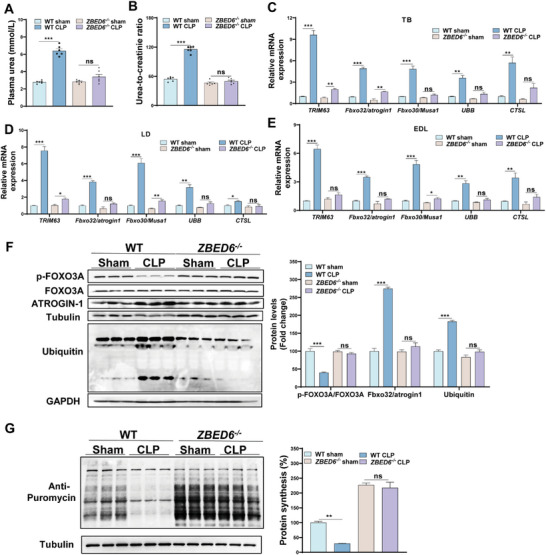
Loss of ZBED6 inhibits protein breakdown during sepsis‐induced muscle atrophy in pigs. A,B) Plasma urea concentration (A) as well as urea‐to‐creatinine ratio (B) in sham or modified CLP pigs 14 days post‐surgery. ZBED6‐deficient pigs (CLP, *n* = 6; sham, *n* = 6), WT (CLP, *n* = 6; sham, *n* = 6). C–E) Relative mRNA expression of markers for atrophy in TB (C), LD (D), and EDL (E) muscles. F) Western blot analysis of muscle atrophy genes in EDL muscles from sham and CLP pigs. (Right panel) Quantification of the p‐Foxo3A/Foxo3A, MURF1, and lysine 48 poly‐ubiquitinated protein levels against internal control α‐Tubulin. G) Representative images (Left) and quantification (Right) for SUnSET puromycin incorporation assay in muscles from sham and CLP pigs 14 days after surgery. ZBED6‐deficient pigs (CLP, *n* = 3; sham, *n* = 3), WT (CLP, *n* = 3; sham, *n* = 3). Data are expressed as mean ± SEM; *p < 0.05, **p < 0.01, ***p < 0.001.

The maintenance of skeletal muscle mass is determined by the balance between anabolic and catabolic processes.^[^ ^23^ ^]^ To investigate whether ZBED6 deficiency affects this balance, we analyzed plasma levels of urea and creatinine, two biochemical markers associated with muscle catabolism, in pigs. Markedly higher levels of urea and urea‐to‐creatinine ratio were shown in septic WT pigs than in their sham controls (**Figure** **4**A,B), whereas these parameters were not elevated in septic *ZBED6^−/−^* pigs, suggesting a protection of ZBED6 deficiency against sepsis‐induced muscle catabolism. In line with this, the expression levels of atrophy markers tripartite motif containing 63 (*TRIM63*), *Fbxo32/atrogin1*, *Fbxo30/Musa1*, ubiquitin B (*UBB*), and cathepsin L (*CTSL*) were strongly induced in TB, LD, and EDL muscles of septic WT pigs (Figure 4C‐E), whereas most of these markers showed no elevations in septic *ZBED6^−/−^* pigs. Forkhead box O3 (FOXO3) is a transcription factor which regulates the transcription of muscle atrophy‐related genes, such as *TRIM63* and *Fbxo32/atrogin1*.^[^ ^24^ ^]^ It is well recognized that phosphorylation inactivates FOXO3 and thus reduces the expression of its target genes. In agreement with this, phosphorylation of FOXO3 was reduced in septic WT pigs (Figure 4F), but not in septic *ZBED6^−/−^* pigs. Elevated protein levels of Fbxo32/atrogin1 and ubiquitin, two downstream signaling components of FOXO3, were shown in septic WT pigs, but not in septic *ZBED6^−/−^* pigs (Figure 4F), corroborating the resistance to sepsis‐induced muscle catabolism upon ZBED6 deficiency. We also measured protein synthesis rate by surface sensing of translation (SUnSET) analysis and found significant reduction in skeletal muscles of septic WT pigs while preserved ability in those of septic *ZBED6^−/−^* pigs (Figure 4G). Together, these results indicate that ZBED6 ablation rescues sepsis‐induced muscle atrophy by maintaining the dynamic equilibrium between protein degradation and synthesis.

Inflammation is one of the main drivers of sepsis‐induced muscle atrophy.^[^ ^2^, ^25^ ^]^ Here, we found that both septic WT and *ZBED6^−/−^* pigs developed persistent fever (Figure S4A, Supporting Information), leukocytosis, and neutrophilia (Figure S4B,C, Supporting Information), as well as similar mRNA levels of inflammatory marker genes IL‐6, TNF‐a, NLRP3, and TLR4 in skeletal muscle (Figure S4D–F, Supporting Information). These results demonstrated that ZBED6 expression is not related to inflammatory, excluding a potential contribution of attenuation of systemic or skeletal muscle specific inflammation to the protection of sepsis‐induced muscle atrophy by ZBED6 deletion.

### 2.6 ZBED6 Controls the Transcription of a Muscle Atrophy Regulator DOCK3

**Figure 5:**
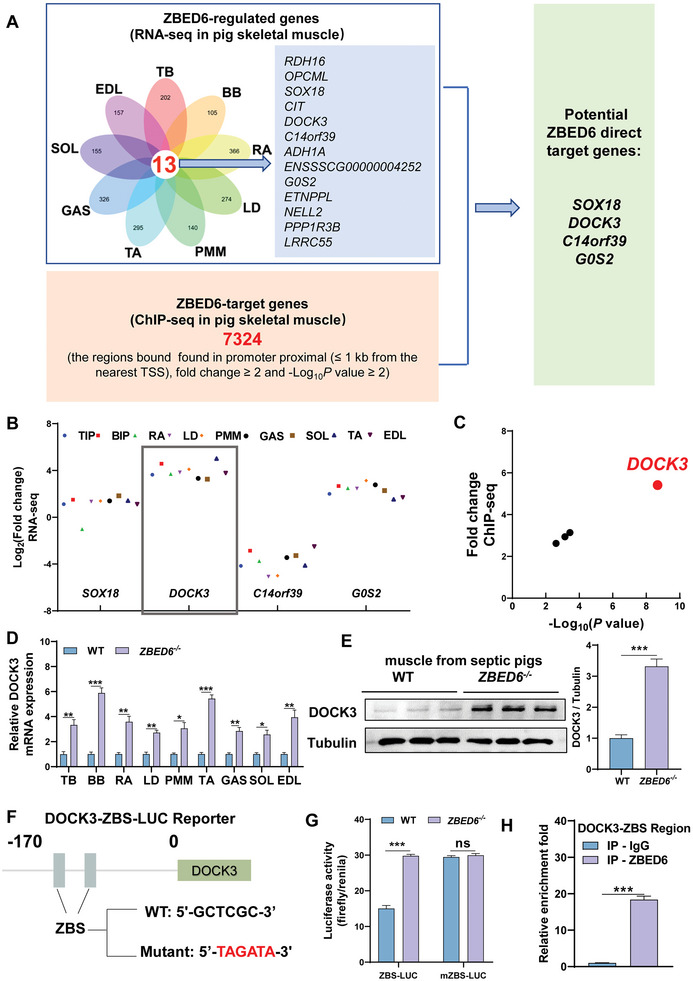
ZBED6 controls DOCK3 transcription in sepsis‐induced muscle atrophy. A) Integrated analysis of RNA‐seq data from nine depots of skeletal muscles of septic WT and ZBED6‐deficient pigs and ZBED6 ChIP‐seq data from pig muscle tissue reveals four potential targets for ZBED6. B) Expression fold change values (Log_2_ of TPM) of *SOX18*, *DOCK3*, *C14orf39*, and *G0S2* in the RNA‐seq analysis from nine depots of muscles of septic WT and ZBED6‐deficient pigs. Triceps brachii; TB, Biceps brachii; BB, Rectus abdominis; RA, Longissimus dorsi muscle; LD, Psoas major muscle; PMM, Gastrocnemius; GAS, Tibialis anterior; TA, Extensor digitorum lateralis; EDL, Soleus; SOL. C) Plot (significance vs fold change) of significantly putative ZBED6 target genes (|fold change |≥ 2 and ‐Log_10_P value ≥ 2) between WT and ZBED6‐deficient pigs. D) DOCK3 mRNA expression in the 9 depots of skeletal muscles of septic WT and ZBED6‐deficient pigs. E) DOCK3 protein expression in the EDL muscle of septic WT and ZBED6‐deficient pigs. F) The wildtype and ZBS mutant sequences are indicated. Cell‐based reporter assays were performed in pig primary satellite cells of ZBED6‐deficient and WT pigs transfected with the wildtype or ZBS mutant luciferase reporter. G) Luciferase analysis showing the effects of ZBED6 on wild‐type DOCK3‐ZBS luciferase (ZBS‐LUC) or mutant DOCK3‐ZBS luciferase (mZBS‐LUC). Data were calculated from three independent replicates. H) ChIP analysis of ZBED6 on DCOK3‐ZBS promoter region was performed in gastrocnemius muscle. Data were calculated from three independent replicates. Data are expressed as mean ± SEM; *p < 0.05, **p < 0.01, ***p < 0.001.

To explore the mechanism underlying ZBED6‐regulated sepsis‐induced muscle atrophy, we compared the transcriptomes of nine anatomically distinct skeletal muscle tissues throughout the body (two in the forelimb, three in the trunk, and four in the hindlimb) from septic *ZBED6^−/−^* pigs and WT controls (Figure S5A–C, Supporting Information), and identified 13 genes with significant expression changes between *ZBED6^−/−^* pigs and WT controls across nine muscles (**Figure** **5**A). Meanwhile, given that ZBED6 is a transcription factor, we also performed ChIP‐seq analysis in pig skeletal muscle to investigate the direct target of ZBED6 and summarized the potential genes bound by ZBED6 in their proximal promoters (≤1 kb from the nearest TSS) in Table S2, Supporting Information. Integrated analysis of the Chip‐seq and RNA‐seq revealed four potential target genes SRY‐box transcription factor 18 (*SOX18*), *DOCK3*, chromosome 14 open reading frame 39 (*C14orf39*), and G0/G1 switch 2 (*G0S2*) for ZBED6 (Figure 5A, left panel). Among these genes, *DOCK3*, with the highest fold change values in RNA‐seq from the nine depots of muscles (Figure 5B) and in ZBED6 ChIP‐seq from the pig muscles (Figure 5C), was identified as a highly potential candidate target for ZBED6. qPCR in nine depots of the skeletal muscles (Figure 5D) and Western blot validation (Figure 5E) showed dramatically increased DOCK3 levels in skeletal muscles of septic ZBED6‐deficient pigs.

To experimentally assess whether *DOCK3* is regulated by ZBED6, we analyzed the promoter region of porcine *DOCK3* and characterized the conserved ZBED6 binding site “GCTCGC” (ZBS) (Figure S6A). A DOCK3 promoter fragment extending from −170 to +392 was used to generate the DOCK3‐luciferase reporter. In transfection assays performed in pig primary satellite cells, reporter activity was induced ≈4‐fold in the absence of ZBED6 (Figure S6B, Supporting Information). Analysis of a series of 5′ deletion mutants showed that truncation of the DOCK3 promoter to 0 resulted in a great loss of the ZBED6 response (Figure S6B). Based on this, we generated two luciferase reporters driven by wildtype and ZBS mutant DOCK3 promoter (Figure 5F). In wildtype ZBS‐LUC construct, ZBED6 deficiency significantly enhanced DOCK3 promoter‐reporter activity (Figure 5G), whereas no difference was shown between ZBED6‐dificient satellite cells and those of WT controls in the mutant form of ZBS. In addition, ChIP‐PCR showed direct binding of ZBED6 to DOCK3 in the skeletal muscle of septic pigs (Figure 5H). Together, these results show that ZBED6 represses the expression of DOCK3 by direct binding to its promoter.

### 2.7 *DOCK3* is Essential for *ZBED6* Deficiency‐Mediated Protection Against Sepsis‐Induced Muscle Atrophy

**Figure 6:**
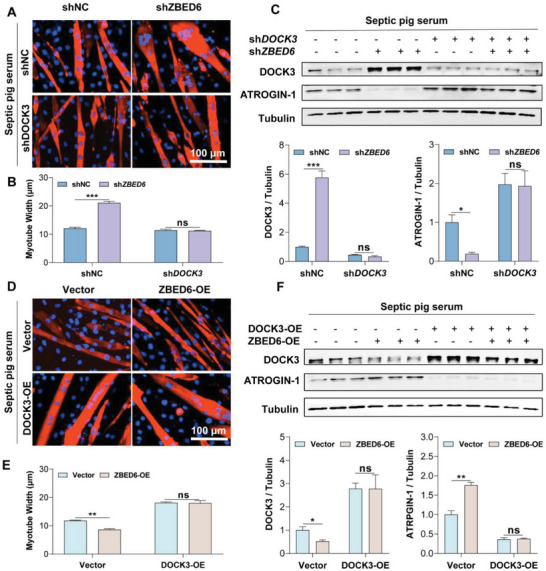
DOCK3 is essential for the protective effect of ZBED6 depletion on sepsis‐induced myotube atrophy. A) Representative images of ZBED6‐depleted myotubes with *DOCK3* knockdown. Myotubes were treated with septic serum. Red indicates myosin heavy chain (MYHC) immunofluorescent staining. Blue indicates DAPI staining of nuclei. Scale bars, 100 µm. B) Quantification for fiber diameter in myotubes described in A. C) Western blot (top) and relative quantification (bottom) of DOCK3 and ATROGIN‐1. D) Representative images of ZBED6‐overexpressing myotubes with DOCK3 overexpression. Scale bars, 100 µm. E) Quantification for fiber diameter in myotubes described in D. F) Western blot (top) and relative quantification (bottom) of DOCK3 and ATROGIN‐1. Data are expressed as mean ± SEM; *p < 0.05, **p < 0.01, ***p < 0.001.

To test whether *DOCK3* plays a major role in *ZBED6* depletion mediated‐resistance against muscle atrophy under septic condition, we established a primary myotube culture system, which was widely used for in vitro muscle atrophy study.^[^ ^25^ ^]^ Compared with normal controls, knockdown of *ZBED6* protected sepsis‐induced myotube atrophy (**Figure** **6**A,B) and reduced the protein expression of ATROGIN‐1 (Figure 6C), accompanied with high protein levels of DOCK3 (Figure 6C). Knockdown of *DOCK3* in myotubes abolished the resistance to sepsis‐induced myotube atrophy by *ZBED6* shRNAs, showing reduced myotube diameter (Figure 6A,B) and elevated ARTOGIN‐1 (Figure 6C), suggesting that *ZBED6* knockdown protects myotubes from sepsis‐induced atrophy by upregulating DOCK3. In contrast, ZBED6 overexpression in myotubes exacerbated sepsis‐induced muscle atrophy, exhibiting small myotube diameter (Figure 6D,E) and high expression levels of ATROGIN‐1 (Figure 6F), accompanied with reduced levels of DOCK3. DOCK3 overexpression was sufficient to rescue aggravated sepsis‐induced atrophy in ZBED6‐overexpressing myotubes (Figure 6D–F). Together, these results demonstrate that DOCK3 is essential for the protective role of ZBED6 deficiency on sepsis‐induced muscle atrophy.

### 2.8 Loss of ZBED6 Activates DOCK3‐Mediated RAC1/PI3K/AKT Signaling Pathway to Protect Against Sepsis‐Induced Muscle Atrophy

**Figure 7:**
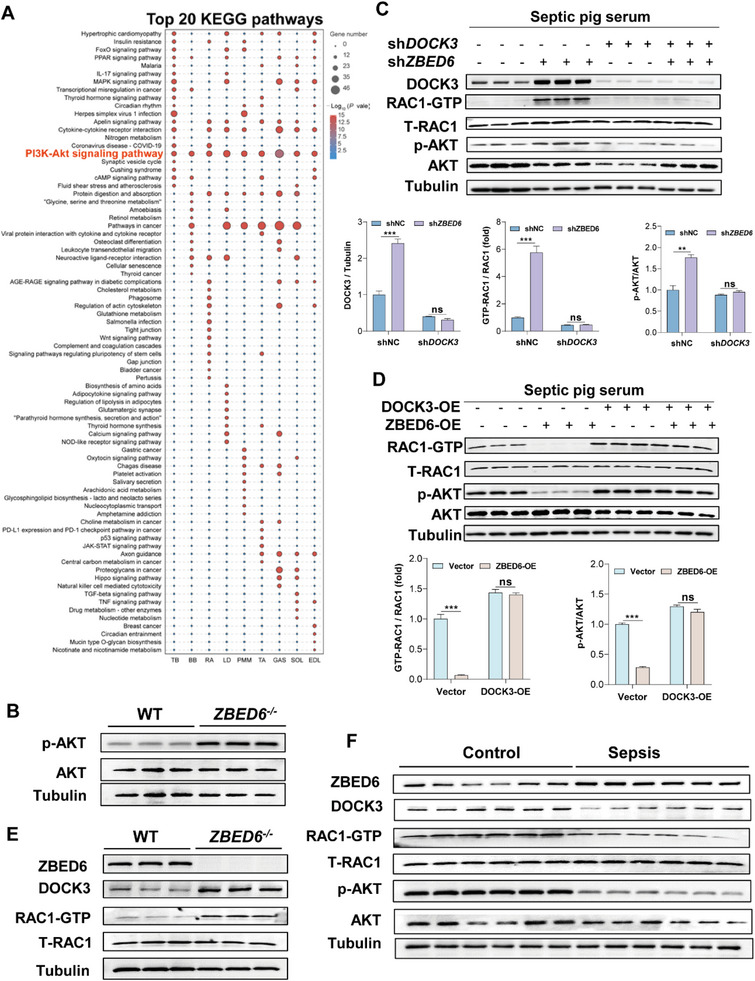
Loss of ZBED6 activates DOCK3‐mediated RAC1/AKT signaling pathway in sepsis‐induced atrophy. A) Top twenty enriched KEGG pathways of DEGs in nine depots of skeletal muscle from septic ZBED6‐deficient pigs compared with WT controls. B) Western blot images showing ZBED6 deficiency results in increased levels of p‐AKT in skeletal muscles of septic pigs. C) Western blot images showing ZBED6 depletion results in increased levels of DOCK3, active Rac1 (Rac1‐GTP), and p‐AKT in pig primary myotubes under normal or septic conditions. Pig primary myotubes infected with control lentivirus (shNC) or ZBED6 shRNA (shZBED6) lentivirus. Seventy‐two hours after infection, myotubes were treated with normal or septic pig serum for 48 h. Western blot (top) and relative quantification (bottom) of DOCK3, Rac1‐GTP, p‐AKT (S473), and AKT. D) Western blot images showing ZBED6 overexpression results in decreased levels of Rac1‐GTP and p‐AKT in pig primary myotubes under normal and septic conditions. Pig primary myotubes infected by adenovirus‐encoding ZBED6 (ZBED6‐OE) or control vector (Vector). Seventy‐two hours after infection, myotubes were treated with normal or septic pig serum for 48 h. Western blot (top) and relative quantification (bottom) of Rac1‐GTP, p‐AKT (S473), and AKT. E) Western blot images showing ZBED6 deficiency results in increased levels of DOCK3 and active Rac1 (Rac1‐GTP) in skeletal muscles of septic pigs. F) Western blot images showing increased levels of ZBED6 and decreased levels of DOCK3, Rac1‐GTPand p‐AKT in skeletal muscles of sepsis patients. Data are expressed as mean ± SEM; *p < 0.05, **p < 0.01, ***p < 0.001.

To further determine the downstream pathway of ZBED6, we compared the transcriptome of the skeletal muscles in septic *ZBED6^−/−^* pigs and WT controls, and found that the genes enriched in PI3K/AKT signaling pathways were consistently upregulated across nine distinct skeletal muscle tissues in septic *ZBED6^−/−^* pigs compared with their WT controls (**Figure** **7**A). Western blot validation revealed elevated phospho‐Akt (p‐AKT) in skeletal muscle of septic ZBED6‐deficient pigs (Figure 7B), confirming a link between ZBED6 and PI3K/AKT pathway.

To further determine whether activation of PI3K/AKT pathway by ZBED6 deficiency is DOCK3 dependent, we knockdown ZBED6 in myotubes and found markedly increased p‐AKT (Figure 7C), accompanied with high protein levels of DOCK3 (Figure 7C). Combined knockdown of *DOCK3* and ZBED6 in myotubes abolished the elevated p‐AKT due to *ZBED6* shRNAs (Figure 7C). Conversely, overexpression of ZBED6 in myotubes decreased the p‐AKT (Figure 7D), with reduced levels of DOCK3 (Figure 6F). Overexpression of DOCK3 restored the p‐AKT in ZBED6‐overexpressing myotubes (Figure 7D). These results suggest that DOCK3 mediates the effect of ZBED6 on the activation of PI3K/AKT pathway.

It has been reported that DOCK3 is an essential activator of RAC1^[^ ^19^, ^26^ ^]^ and RAC‐GTP, the active form of RAC1,^[^ ^26^, ^27^ ^]^ is able to activate the PI3K/AKT pathway.^[^ ^28^ ^]^ We thus measured RAC1‐GTP and the activation of the pathway, showing increased protein levels of RAC1‐GTP and p‐AKT in ZBED6 knockdown myotubes (Figure 7C), whereas knockdown of DOCK3 markedly decreased protein levels of RAC1‐GTP and p‐AKT (Figure 7C). In contrast, in ZBED6‐overexpressing myotubes, decreased protein levels of RAC1‐GTP and p‐AKT were observed, whereas overexpression of DOCK3 completely restored these changes (Figure 7D). These results suggest that ZBED6 downregulates DOCK3/RAC1/PI3K/AKT. Consistent with these cellular results, skeletal muscles from septic ZBED6‐deficient pigs displayed increased protein levels of DOCK3, RAC1‐GTP (Figure 7E), and activation of PI3K/AKT pathway (Figure 7B). In contrast, skeletal muscles from sepsis patients with muscle atrophy showed significantly increased protein levels of ZBED6 and reduced levels of DOCK3/RAC1‐GTP/p‐AKT (Figure 7F). These results show that ZBED6 deficiency activates the DOCK3/RAC1/PI3K/AKT signaling pathway which is required for its resistance to sepsis‐induced muscle atrophy.

## 3 Discussion

Sepsis‐induced muscle atrophy is a devastating condition that greatly affects the quality of life of survivors^[^ ^29^ ^]^ and increased the costs for the health care system. In this study, we identified ZBED6 as a potential target for sepsis‐induced muscle atrophy by creating a modified septic pig model with CLP procedure. We validated this conclusion in ZBED6‐deficient pigs and human patients diagnosed with sepsis‐induced muscle atrophy. We show a positive correlation between ZBED6 expression and muscle atrophy, as well as depletion of ZBED6 protects against sepsis‐induced muscle atrophy. Mechanistically, integrated transcriptomic and ChIP‐seq analysis revealed DOCK3 as the direct target of ZBED6 and RAC1/PI3K/AKT as its downstream signaling pathway. These findings provide novel insights into the molecular mechanisms underlying sepsis‐induced muscle atrophy and may have implications for the development of novel therapies targeting ZBED6 for sepsis.

Since its identification in 2009 as a transcription factor, ZBED6 has been reported to regulate the growth of skeletal muscle in placental mammals under physiological conditions.^[^ ^13^ ^]^ In mice and pigs, deficiency of ZBED6 increased growth rate and skeletal muscle mass.^[^ ^13^, ^14^ ^]^ Our finding that ZBED6 regulates sepsis‐induced muscle atrophy extends its role from physiological conditions to a type of muscle pathology, expanding the function and potential application of ZBED6. It is worth noting that muscle atrophy occurs in various conditions, such as aging,^[^ ^30^ ^]^ cancer‐associated cachexia,^[^ ^31^ ^]^ and diabetes.^[^ ^32^ ^]^ Whether ZBED6 also involves in the muscle loss under these conditions merits further investigation. Furthermore, our previous study showed a broad tissue distribution of ZBED6, and deficiency of ZBED6 affects multiple internal organs including heart and liver.^[^ ^14a^ ^]^ This opens a range of relevant questions in sepsis where multiple organs are affected. Whether ZBED6 also contributes to the multiorgan injuries induced by sepsis is another fertile area for future research.

ZBED6 is a transcription factor which could regulate multiple gene expression. Different from its first identified target IGF2 under normal skeletal muscle growth, we conclude that DOCK3 is the main target of ZBED6 under conditions of sepsis‐induced muscle atrophy based on the evidence that 1) integrated analysis of RNA‐seq in nine depots of skeletal muscles from septic *ZBED6^−/−^* pigs and WT controls and ZBED6 ChiP‐seq in pig muscle tissue converged on DOCK3, 2) direct binding of ZBED6 on the promoter region of DOCK3, and 3) cellular phenotype that knockdown of DOCK3 abolished the protective role of ZBED6 deficiency on sepsis‐induced muscle atrophy. Previous studies have shown a key role of DOCK3 in the pathogenesis of skeletal muscle atrophy. In humans, loss‐of‐function variants in DOCK3 cause developmental delay, muscle hypotonia, and ataxia.^[^ ^20^ ^]^ In mice, global Dock3 deficiency induced muscle atrophy and impaired muscle function.^[^ ^19b^ ^]^ Dock3‐deficient myoblasts show defective myogenic differentiation.^[^ ^19b^ ^]^ However, in Duchenne muscular dystrophic mice, haploinsufficiency of Dock3 improved dystrophic muscle pathologies. Our study extended a protective role of DOCK3 in sepsis‐induced muscle atrophy and elucidated its upstream transcription factor ZBED6 and downstream RAC1/PI3K/AKT pathway, revealing a novel regulatory mechanism of DOCK3 underlying the occurrence of muscle atrophy.

One key implication of our work is the creation of a novel pig CLP model that better recapitulates the human septic response. Animal models used for studying the pathophysiology of sepsis are categorized into three main classes: toxemia models, bacterial infection models, and host‐barrier disruption models.^[^ ^33^ ^]^ Among them, host‐barrier disruption models, particularly the CLP model, are considered the gold standard for sepsis study due to their similarity to the progression of human sepsis.^[^ ^33^, ^34^ ^]^ In this study, by changing the surgical strategy from cecal ligation and puncture to cecal incision and interrupted sutures, we developed a novel sepsis pig CLP model that overcomes the shortcomings of the traditional pig CLP model for their high acute mortality rate, thus providing more flexibility in investigating the onset and progression of sepsis to multi‐organ failure. The novel pig sepsis model recapitulates the multi‐organ pathological changes and tissue damages observed in human sepsis across a range of spatiotemporal scales. This model could be explored for understanding the pathological mechanisms of sepsis, for developing effective treatments for sepsis, for evaluating the pharmacokinetics and pharmacodynamic effects of potential therapeutic agents before clinical translation. In addition, this model is also useful for evaluating the safety and efficacy of novel medical devices, such as medical robotics, implantable wearables, and flexible three‐dimensional‐printed medical devices, that can be used for sepsis patient care, treatment, and monitoring. Overall, the modified pig CLP model holds great promise for advancing our understanding of sepsis pathogenesis and improving clinical outcomes for sepsis patients.

One limitation of our investigation is the absence of evidence regarding the effects of ZBED6 deficiency, apart from ameliorating sepsis‐triggered muscle atrophy, on the dysfunction of other organs that are often affected during sepsis. Further research is necessary to explore the systemic role of ZBED6 in sepsis. In addition, since systemic ZBED6‐deficient pigs were used in this study, our study cannot rule out the possibility that the observed effects of ZBED6 deficiency on the protection against sepsis‐induced muscle atrophy may be partially attributed to its actions in other organs besides skeletal muscle.

## 4 Conclusion

Our findings suggest that ZBED6 is a promising therapeutic target for sepsis‐induced muscle atrophy, with potential clinical applications for the treatment of muscle loss in sepsis. Furthermore, our innovative sepsis pig model serves as a powerful tool for facilitating mechanistic exploration of sepsis‐induced muscle wasting and other related multi‐organ injuries, as well as for developing and testing potential therapeutics, thus providing an idea experimental model for pre‐clinical sepsis studies.

## 5 Experimental Section

### Human Studies

Muscle biopsies and blood samples from sepsis patients with muscle atrophy were collected from the Tang Du Hospital. The cohort included 25 sepsis patients with muscle atrophy and 15 orthopedic patients who were used as controls. The Ethics Institutional Review Board of Tang Du Hospital, Fourth Military Medical University approved all human studies conducted in this research, with clinical ethical approval (K202301‐09). In compliance with the principles of the Declaration of Helsinki and under the supervision of the ethics committee of Tang Du Hospital of Fourth Military Medical University, human rectus femoris muscle and blood samples were collected and utilized with informed consent obtained from all participants. Sepsis was determined using Sepsis‐3 criteria, with organ dysfunction defined as a SOFA score of 2 or more points.^[^ ^35^ ^]^ In this study, all patients included underwent an initial ultrasound examination within 40 h of their admission to the intensive care unit (ICU). Follow‐up ultrasound images were obtained on day 7 to evaluate the extent of muscle loss. Muscle loss was assessed based on the comparison of the rectus femoris cross‐sectional area (RF‐CSA) obtained by the ultrasound examination from these two‐time points as previously described.^[^ ^4^, ^36^ ^]^ Patients were categorized into two groups: atrophy and non‐atrophy, based on the percentage of RFCSA loss. Specifically, patients with a loss of more than 10% in RFCSA were classified as having muscle atrophy based on previously established threshold.^[^ ^4b^ ^]^ Muscle biopsies were immediately frozen in liquid nitrogen and stored at −80 °C until further analysis. Blood samples were collected in EDTA tubes (BD Vacutainer) to separate plasma and stored at −80 °C until further analysis.

### Animals

Animal studies were conducted with approval from the Institutional Animal Care and Use Committee of Northwest A and F University. Bama miniature pigs were bred and raised at the Laboratory Animal Center of Chengdu Clonorgan Biotechnology Co. LTD, where they were given ad libitum access to diet and water, unless otherwise specified. Male offspring were genotyped and randomly assigned at 9 months of age, with littermate controls employed in all cases. To maintain objectivity, all experiments were performed by investigators who were blinded to the experimental groups.

### Animals—Traditional Porcine Model of Sepsis

The conventional porcine model for studying sepsis was established utilizing the cecal ligation and puncture (CLP) surgical method as previously documented.^[^ ^37^ ^]^ In brief, pigs were subjected to anesthesia and induced with peritonitis via cecal ligation and puncture. Following resuscitation, the animals were relocated to their designated housing area with ad libitum access to food.

### Animals—Modified Porcine Model of Sepsis

The porcine model of sepsis was modified by implementing two main changes: 1) Utilizing a novel surgical technique involving cecal incision and interrupted sutures under general anesthesia and 2) administering supportive therapeutic interventions following the surgical procedure. The surgical procedure involved creating a small incision at approximately 8 cm midline laparotomy to expose the cecum and terminal ileum. Prior to cecal ligation and puncture, intestinal content was rubbed into the cecum from the ascending colon. A 5 cm incision was then made below the ileocecal valve to puncture the cecum and permit fecal matter to extrude into the peritoneum. The cecum was ligated using interrupted sutures at 0.5 cm intervals with a nonabsorbable silk suture. The abdominal cavity was subsequently closed by suturing the peritoneal and skin layers, disinfecting with iodine, and covering the incision site with antibiotic ointment and light compressive dressings. Sham‐operated pigs underwent an identical procedure without the CLP.

During surgery, preheated lactate ringer solution (10 ml kg^−1^; 37 °C) was intravenously administered to compensate for body fluid loss, while a 5% dextrose solution (10 ml kg^−1^; 37 °C) was also administered to prevent hypothermia and hypoglycemia.^[^ ^34^, ^38^ ^]^ Postsurgery, animals were treated with analgesic and anti‐inflammatory medication (carprofen, Rimadyl, 4 mg kg^−1^, i.m.) once daily for at least 4 days.^[^ ^39^ ^]^ Regular monitoring of survival, body weight, and body temperature was carried out. On the 14th day following CLP, sepsis survivors and non‐sepsis controls were euthanized using established methods.^[^ ^38^, ^39^ ^]^

### Animal Protocol 1: Traditional and Modified CLP Porcine Model of Sepsis

Eighteen adult minipigs (9‐month‐old males) with a mean weight of 33.07 kg were included in this experiment. The animals were randomly divided into three groups: sham controls (*n* = 6), traditional CLP (*n* = 6), and modified CLP (*n* = 6). Inhalation of 5% isoflurane was used to anesthetize the animals, and laparotomy was performed while maintaining anesthesia at 2.5%. Body weight and temperature were monitored daily. On the 14th day following CLP, sepsis survivors and nonsepsis controls were euthanized, as previously described. Blood was collected via the jugular vein into EDTA‐coated tubes, and plasma samples were separated by centrifugation at 3,000 rpm at 4 °C for 10 min. Organs and tissues were collected, weighed, and frozen at −80 °C until further analysis.

### Animal Protocol 2: Modified CLP WT and ZBED6‐Deficient Porcine Model of Sepsis

Twenty‐four adult minipigs (9‐month‐old males) with a mean weight of 37.6 kg were included in this experiment. The WT and ZBED6‐deficient pigs were separately randomized and divided into four groups: WT sham controls, WT CLP, ZBED6‐deficient sham controls, and ZBED6‐deficient CLP. N = 6 for each group. Inhalation of 5% isoflurane was used to anesthetize the animals, and laparotomy was performed while maintaining anesthesia at 2.5%. On the 14th day following CLP, blood was collected into EDTA‐coated tubes, and plasma was obtained as in Animal Protocol 1. Organs and tissues were carefully collected, weighed, and frozen at −80 °C until subsequent analysis.

### Immunostaining and Histology

For human studies, the examination of hematoxylin and eosin (HE) staining was conducted on paraffin‐embedded muscle tissue sections for histology and morphometry.^[^ ^40^ ^]^ For pig studies, cryo‐sections (8 µm) of frozen muscles were processed for HE staining and immunohistochemistry using standard procedures for histology and morphometry.^[^ ^41^ ^]^ Muscle fiber type imaging was performed on the frozen sections by staining them with primary monoclonal antibodies against type I myosin heavy chain (MHC) (BA‐D5, mouse IgG2b), type IIA MHC (SC‐71, mouse IgG1), and type IIB MHC (BF‐F3, mouse IgM), at a dilution of 1:400. The primary monoclonal antibodies were then detected using goat anti‐mouse secondary antibodies against IgG2b (Alexa 568, A‐21144, Invitrogen), IgG1 (Alexa 488, A‐21121, Invitrogen), and IgM (Alexa 405, ab175662, Abcam). The stained sections were photographed using a digital pathology slide scanner (VS120‐S6‐W, Olympus Corporation, Tokyo, Japan), and fiber cross‐sectional areas were measured for myofiber size. Additionally, different fiber types, fiber count, and type percentages were assessed using Image‐Pro Plus software.

### In Vivo Protein Synthesis Rate Measurements

In vivo protein synthesis rates were measured via the SUnSET technique.^[^ ^42^ ^]^ 9‐monthe‐old male WT and ZBED6‐deficient pigs (6 pigs per group) were intraperitoneal injected with 0.04 mmol g^−1^ puromycin. Thirty‐minute postinjection, muscles were collected and frozen in liquid Nitrogen for immunoblot analysis using antipuromycin antibody (clone 12D10, Sigma).

### Primary Myoblast Purification and Culture

Cell isolation and culture MuSC‐derived primary myoblast isolation was performed as previously described.^[^ ^43^ ^]^ In some in vitro experiments, serum was obtained from septic animals at 14 days post‐CLP by internal jugular vein puncture and centrifugation at 500 g for 15 min. The resulting supernatant was used to replace FBS in the culture medium, while the remainder of the medium remained unchanged as previously described.^[^ ^4a^ ^]^

### RNA Interference

Scrambled shRNA, ZBED6, and DOCK3 shRNAs were obtained from Hanheng Biotechnology (Hanheng Biotechnology Co., Ltd., Shanghai, China). The sequences of ZBED6 and DOCK3 shRNAs were listed in Table S3, Supporting Information.

### Immunofluorescence and Cell Differentiation Measurements

Immunofluorescence experiments and cell morphology measurements were performed using a previously described protocol.^[^ ^44^ ^]^ The following antibodies were used: mouse anti‐MHC (1:2 MF20, R&D Systems, MAB4470) and anti‐mouse secondary antibody Alexa Fluor 568 conjugated (1:100, Invitrogen, A‐21144). The samples were washed three times and nuclei were counterstained with DAPI (Thermo Fisher Scientific, R37606). Myotube width was analyzed by ImageJ, as previously described.^[^ ^25^ ^]^

### Plasmid Construct

To generate expression vectors for ZBED6 and DOCK3, the coding sequences of pig ZBED6 (NCBI Sequence ID: NM_001394675.1) and DOCK3 (NCBI sequence ID: XM_021068831.1) were inserted into pcDNA3.1 vectors. For overexpression of ZBED6 and DOCK3, adenovirus was utilized as the vector, which was obtained from Hanheng Biotechnology (Hanheng Biotechnology Co., Ltd., Shanghai, China). Knockdown of pig DOCK3 and ZBED6 was achieved using shRNA expression lentiviral vectors purchased from Hanheng Biotechnology (Shanghai, China).

A 499 bp fragment of the DOCK3 promoter was isolated by PCR using primers listed in Table S3, Supporting Information and subsequently digested with KpnI and HindIII. The insertion was then ligated into the pGL3‐basic vector (Promega, Madison, WI, USA) to create the DOCK3 promoter‐reporter plasmid pGL3‐1. After sequencing, pGL3‐1 was used as a template to isolate pGL3‐2 and pGL3‐3 through PCR. The mutated ZBED6‐binding site (ZBS) DNA fragment was synthesized by Qingke Biotechnology Company (Wuhan, China).

### Luciferase Assay

Isolation and culture of primary muscle stem cells from wide‐type and *ZBED6^−/−^* pigs. DOCK3 promoter was amplified from wide‐type pig and cloned into the plasmid pGL3 Basic (carrying firefly luciferase, Promega). Cells were transfected by electroporation with 1 mg of pGL3‐DOCK3 vector, empty vector was served as a control, 50 ng of PRL‐TK vector (carrying Renilla luciferase, Promega) was transfected simultaneously to serve as internal control and plated in 24‐well plates containing DMEM with 10% serum. Thirty‐six hours posttransfection, luciferase activity was measured using the Dual Luciferase Reporter Assay Kit (Promega) and Luminometer (Tecan). Five independent reactions were carried out for each experimental point.

### ChIP–Seq and ChIP‐PCR

Chromatin immunoprecipitation followed^[^ ^14a^ ^]^ by high‐throughput sequencing (ChIP‐seq) and ChIP‐PCR were conducted as previously reported using primers listed in Table S3, Supporting Information.

### Blood Sampling and Hematology

In this study, blood samples were collected from pigs subjected to sham or CLP operation before and after the procedures. Jugular venipuncture was used to collect the blood samples, which were then collected into evacuated tubes containing EDTA. A PE‐6800 VET automated hematology analyzer (PROKAN, Shenzhen, China) is used to assess the complete blood cell count.

### Plasma Chemistry

To analyze the levels of various substances in the plasma of pigs subjected to sham or CLP operation, plasma samples before and after the operations were collected. The collected plasma samples were then separated and subjected to standard biochemical analyses using the biochemical autoanalyzer (Hitachi 7180 automated analyzer, Tokyo, Japan) at the Yang‐ling Demonstration District Hospital of Shaanxi Province. The substances included urea, creatinine, ALB, ALT, and AST were measured. Standard procedures for these analyses were followed.

### Real‐Time Reverse Transcriptase PCR

In this study, real‐time reverse transcriptase polymerase chain reaction (RT‐PCR) was employed to investigate gene expression levels in human and pig muscles. To extract total RNA from the samples, the TRIzol reagent method was used, which had been previously reported. To synthesize complementary DNA (cDNA), the guidelines provided by the cDNA synthesis kit manufacturer were followed. The resulting cDNA was then analyzed via RT‐PCR using a CFX 96 Real‐Time PCR Detection System. Gene expression values using the comparative Ct method (2‐ΔΔCt) were calculated, as outlined in the literature. The primer sequences for the genes used in this study are listed in Table S3, Supporting Information.

### Immunoblotting

In order to investigate the expression of specific proteins, the immunoblotting technique known as western blotting was employed. The method was performed in accordance with a previously reported protocol49. Primary myoblasts and muscles were washed with PBS and then lysed in RIPA lysis buffer (P0013C, Beyotime Biotechnology, Shanghai, PRC). To ensure accurate measurements, all protein levels were normalized to the housekeeping proteins, GAPDH or Tubulin. Densitometric quantification of the western blot‐ting bands was performed using ImageJ software. The antibodies used for protein detection are listed in Table S4, Supporting Information.

### Rac1 Immunoprecipitation/Activity Assays

Rac1‐GTP and total Rac1 levels were determined through immunoprecipitation/activity assays using the Active Rac1 Pull‐Down and Detection Kit, according to the manufacturer's instructions (Thermo Fisher Scientific Inc.). To determine Rac1‐GTP levels in pig myotube cell cultures, 25 micrograms of whole‐cell lysates were used. To determine Rac1‐GTP levels in skeletal muscles of septic WT and ZBED6‐deficient pigs, orthopedic controls, and sepsis patients, 100 µg of total protein tissue lysates were used in the Rac1‐GTP immunoprecipitation assay. For normalization, 25 µg of total protein lysate inputs from both cell and muscle lysates were used to measure the levels of total Rac1 protein and a Tubulin loading control.

### RNA Sequencing and Data Analysis

The RNA‐seq libraries were constructed for the nine depots muscle samples after CLP for each of the WT and ZBED6 KO pigs. Muscle tissue sample was homogenized in a tissue grinder for total RNA extraction using TRIzol (Invitrogen, USA) extraction method. The integrity and concentration of total RNA were evaluated using an Agilent 2100 Bioanalyzer (Agilent Technologies, Palo Alto, CA, USA) and NanoDrop 2000&8000 spectrophotometer (Nanodrop Technologies, Wilmington, DE, USA). rRNA‐depleted and random priming RNA‐seq libraries were constructed and then sequenced on an MGI DNBSEQ platform to produce an average of ≈48 million 150‐bp paired‐end raw reads and ≈47 million high‐quality reads for each library at Annoroad Corporation (Beijing, China). Further analyses were based on high‐quality data. Processed reads from each sample were mapped to the pig reference genome (Sscrofa11.1 with the release‐102 annotations from ENSEMBL database) by the STAR alignment tool (v2.6.0c). And the expression levels were acquired with Kallisto software in TPM (transcripts per kilobase million). The mRNA that an expression value greater than 0.5 TPM in at least one library were considered expressed and were selected for further differential expression analysis. The differential expression analysis was performed using the edgeR package (v 3.22.5), with | (fold change) | >2 and *p* < 0.05 as the cut‐offs for statistical significance. Function enrichment analyses were performed using Metascape (http://metascape.org/gp/index.html#/main/step1) with default parameters. Genes in the pig genome were converted to human orthologs, which was used as inputs for the enrichment. Human (Homo sapiens) was chosen as the target species, and enrichment analysis was performed against all genes in the genome as the background set, with Kyoto Encyclopedia of Genes and Genomes (KEGG) as the ontology source. The top ten most statistically significant terms were selected as the outputs.

### Statistical Analysis

In this study, statistical significance was assessed using two methods. Firstly, for comparisons among multiple groups, one‐way or two‐way analysis of variance with Tukey's multiple comparison tests was performed at the 5% significance level to determine differences in mean values. Secondly, two‐sided Student's *t*‐test was used for statistical analysis of two contrasts. The data were plotted using GraphPad Prism 8 software, with error bars indicating the standard error of the mean. The experiment was conducted in triplicate and repeated in independent experiments. It was considered *p* < 0.05 to be statistically significant. *p < 0.05, **p < 0.01, ***p < 0.001.

## Conflict of Interest

The authors declare no conflict of interest.

## Author Contributions

L.H., D.P., P.L., and D.W. are co‐first authors. H.L., L.J., L.Y., M.L., and J.W. conceptualized the study. H.L., D.P., P.L., D.W., B.X., R.Z., L.J., X.Z., X.X., and J.D. contributed to methodology. H.L., L.J., L.Y., M.L., and J.W. performed investigations. H.L. and P.L. visualized the study. D.P., J.L., and L.J. supervised the study. H.L., P.L., L.Y., M.L., and J.W. wrote the original draft. H.L., L.J., L.Y., M.L., and J.W. reviewed and edited the final manuscript.

## Supporting information

Supporting Information

Supporting Information

## Data Availability

The data that support the findings of this study are available from the corresponding author upon reasonable request.
