# Supplementary material for: Loss of ZBED6 Protects Against Sepsis‐Induced Muscle Atrophy by Upregulating DOCK3‐Mediated RAC1/PI3K/AKT Signaling Pathway in Pigs
Source: Adv Sci (Weinh). 2023 Aug 7;10(29):2302298. doi: 10.1002/advs.202302298 (PMC10582467; doi:10.1002/advs.202302298)
Supplement: Supplementary file 1 — Supporting Information [file ADVS-10-2302298-s002.pdf]

ADVANCED SCIENCE
Open Access

Supporting Information

for *Adv. Sci.*, DOI 10.1002/advs.202302298

Loss of ZBED6 Protects Against Sepsis-Induced Muscle Atrophy by Upregulating DOCK3-Mediated RAC1/PI3K/AKT Signaling Pathway in Pigs

Huan Liu, Dengke Pan, Pu Li, Dandan Wang, Bo Xia, Ruixin Zhang, Junfeng Lu, Xiangyang Xing, Jiaxiang Du, Xiao Zhang, Long Jin, Lin Jiang, Linong Yao*, Mingzhou Li* and Jiangwei Wu**

Supporting Information

Loss of ZBED6 protects against sepsis-induced muscle atrophy by upregulating DOCK3-mediated RAC1/PI3K/AKT signaling pathway in pigs

Huan Liu, Dengke Pan, Pu Li, Dandan Wang, Bo Xia, Ruixin Zhang, Junfeng Lu, Xiangyang Xing, Jiaxiang Du, Xiao Zhang, Long Jin, Lin Jiang*, Linong Yao*, Mingzhou Li*, and Jiangwei Wu*

Fig. S1

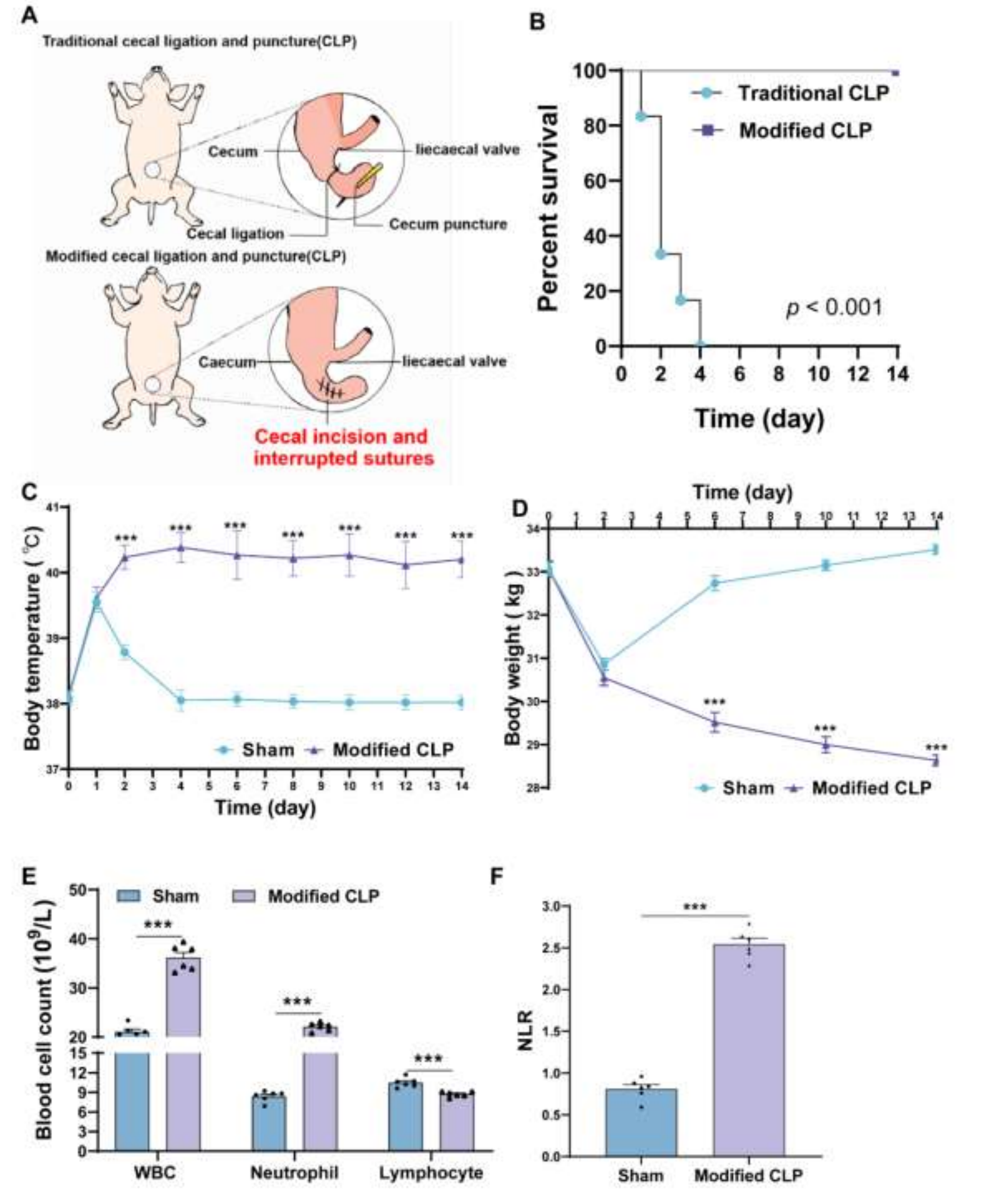

Figure S1. Modified CLP improves survival in a porcine sepsis model. (A) Schematic representation of the traditional and modified surgical procedures for the cecal ligation and puncture (CLP) pig model. **(B)** The Kaplan-Meier survival plot demonstrates the improved survival of pigs following modified CLP (n=6) compared to traditional CLP (n=6) following sham laparotomy. Statistical significance was determined using the log rank test. **(C-D)** Body temperature (C) and body weight (D) were measured in both sham and CLP pigs. **(E-F)** WBC,

neutrophil, and leukocyte counts were quantified (E), along with neutrophil-to-lymphocyte ratio; NLR(F). Data are expressed as mean ± SEM; $*p < 0.05$, $**p < 0.01$, $***p < 0.001$.

Fig. S2

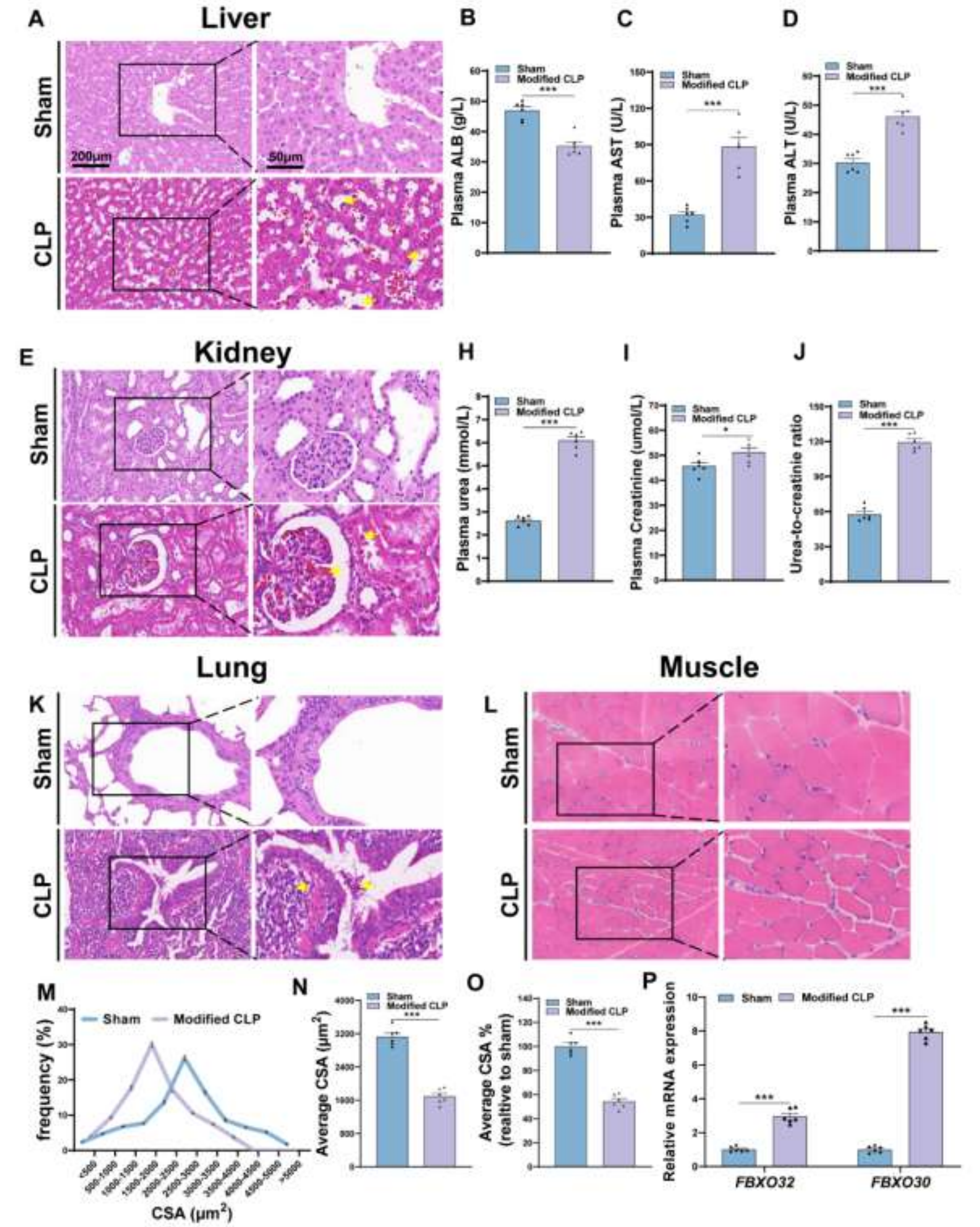

Figure S2. Modified pig sepsis model replicates human sepsis-associated multiorgan damage and muscle atrophy. Bama pigs underwent modified CLP or sham surgery (n=6), and were sacrificed after 14 days. **(A-D)** Modified CLP caused liver injury in pigs, as indicated by hepatic congestion, ballooning hepatocytes, and inflammatory cellular infiltrate observed in HE staining images of liver tissue (A) along with plasma chemistry profile

analysis of albumin (B), AST (C), and ALT (D). **(E-J)** Kidney injury was also shown in modified CLP pigs, with congested glomeruli, increased nuclear staining, and necrotic tubular epithelial cells shown in HE staining images of kidney tissue (E), and plasma chemistry profile analysis of urea (H) and creatinine (I). Urea-creatinine ratio was also determined (J). K. Lung injury was evident in modified CLP pigs with alveolar damage and inflammatory infiltrate observed in HE staining images of lung tissue. **(L-P)** Modified CLP caused muscle atrophy, as shown in HE staining images of skeletal muscle tissue (L). Distribution of myofiber sizes was analyzed from the CSA of ~100 myofibers of each sample (M), and average CSA (N) and average CSA% (O) were quantified and expressed as percent-wise change compared with the respective sham group. qPCR analysis of muscle atrophy maker genes *FBOX32* and *FBOX30* in muscles from sham and CLP pigs (P). The mRNA expression was normalized to GAPDH, and the data are presented as mean ± SEM. *$p < 0.05$, **$p < 0.01$, ***$p < 0.001$.

Fig. S3

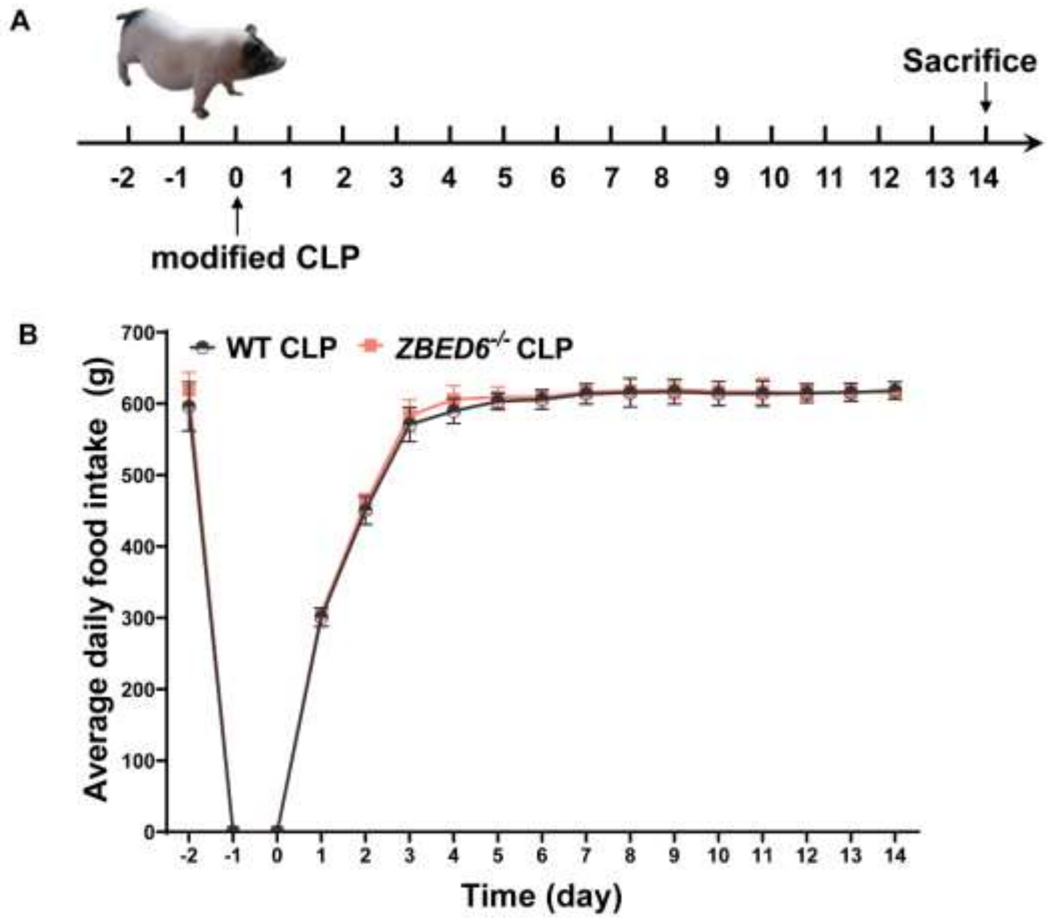

Figure S3. Average daily food intake in ZBED6-deficient pigs and WT controls before and after modified CLP. (A) Schematic diagram of pig treatment. **(B)** Average daily food intake.

Fig. S4

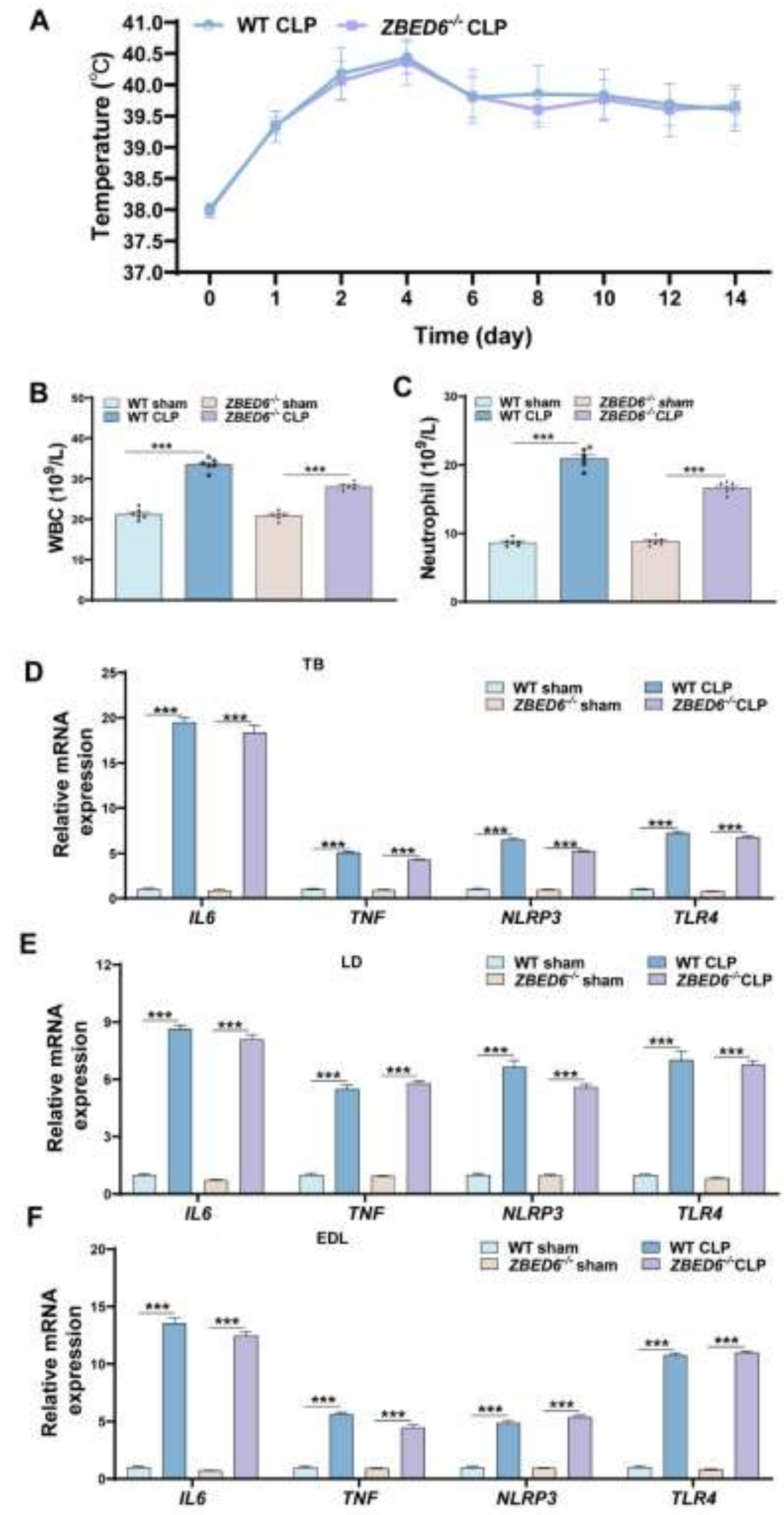

Figure S4. ZBED6 expression is not related to inflammation. Nine-month-old ZBED6-deficient pigs and WT pigs were subjected to modified CLP or sham surgery. After 14 days, the pigs were sacrificed. **(A)** Body temperature was determined before the surgery and daily for the following 14 days. **(B-C)** Blood hematologic results of pigs before surgery and 14

days after surgery. White blood cell (WBC) count (B) and neutrophil count (C). **(D-F)** mRNA expression of inflammation-related genes interleukin-6 (IL-6), tumor necrosis factor (TNF)-α, nucleotide-binding domain, leucine-rich repeat containing protein (NLRP3) and toll-like receptor 4 (TLR4) in TB (D), LD (E) and EDL (F) muscles of pigs. mRNA expression was normalized to GAPDH. ZBED6-deficient pigs (CLP, n = 6; sham, n = 6), WT (CLP, n = 6; sham, n = 6). Data are presented as mean ± SEM. *$p < 0.05$, **$p < 0.01$, ***$p < 0.001$. TB, Triceps brachii; LD, Longissimus dorsi; EDL, Extensor digitorum lateralis.

Fig. S5

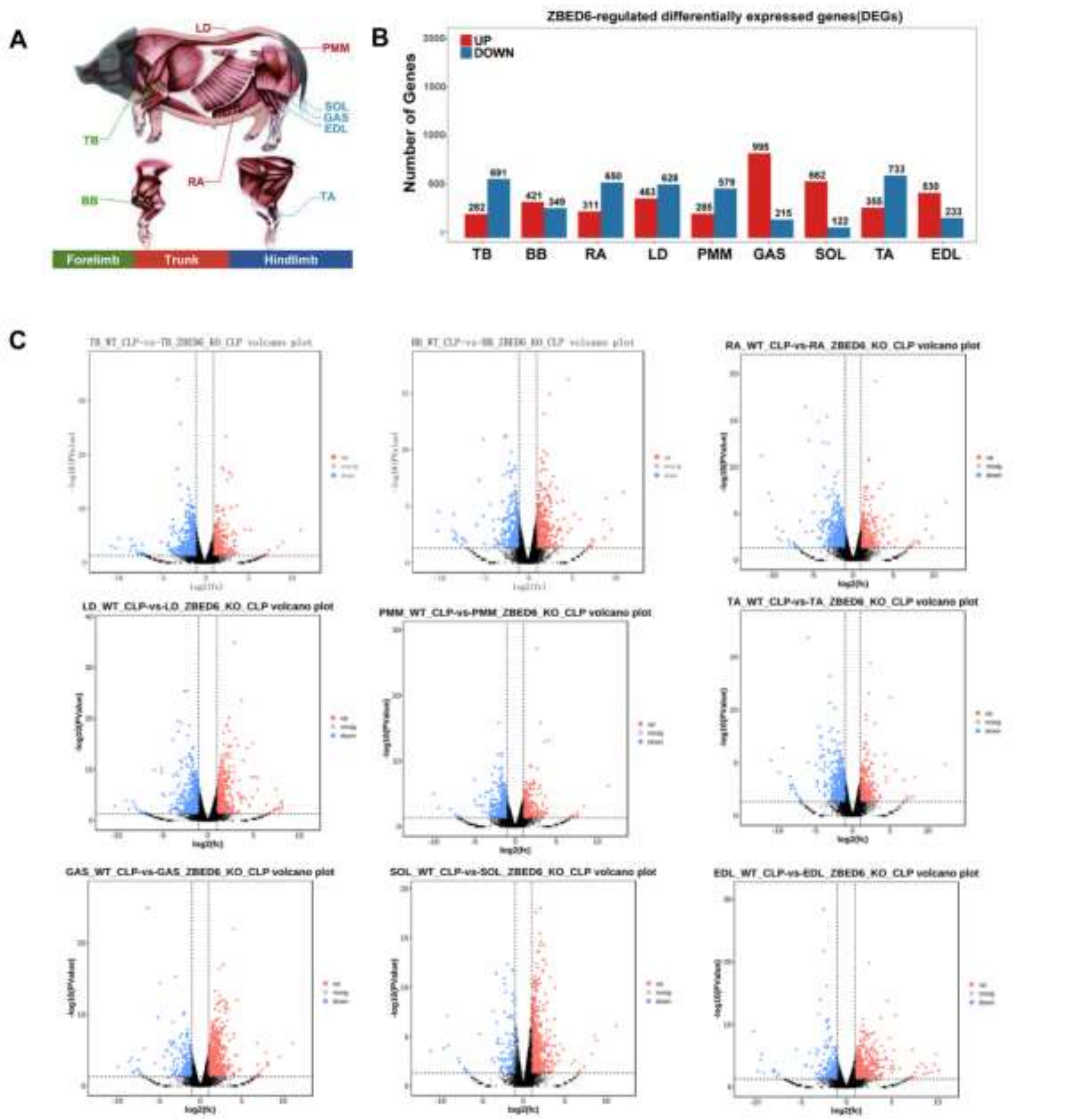

Figure S5. The transcriptomes of skeletal muscles from septic WT and ZBED6-deficient pigs. (**A**) Illustration of the anatomical position of nine depots of skeletal muscles. (**B**) Bar graphs representing differential gene expression numbers of the 9 depots of skeletal muscles samples from septic WT and ZBED6-deficient pigs. (**C**) Volcano plots showing DEGs (|fold change| > 2, p<0.05). Triceps brachii; TB, Biceps brachii; BB, Rectus abdominis; RA, Longissimus dorsi muscle; LD, Psoas major muscle; PMM, Gastrocnemius; GAS, Tibialis anterior; TA, Extensor digitorum lateralis; EDL, Soleus; SOL. Data are expressed as mean ± SEM; *$p < 0.05$, **$p < 0.01$, ***$p < 0.001$.

Fig. S6

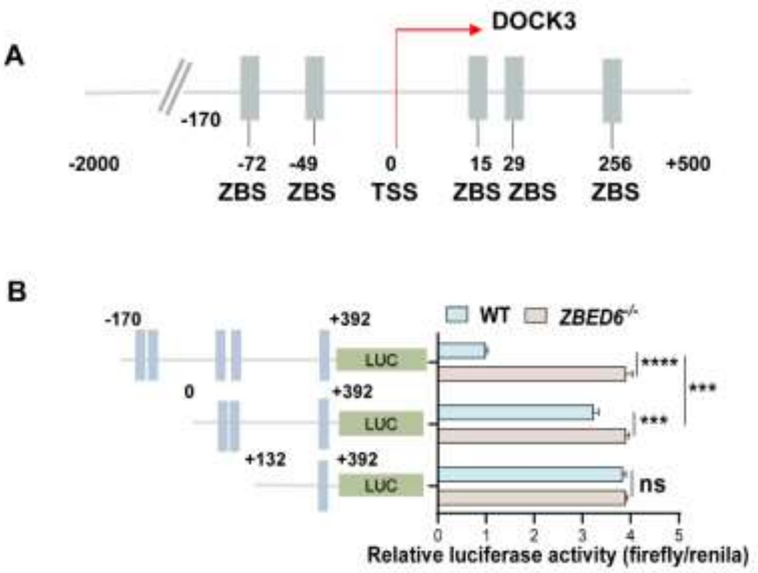

Figure S6. The transcriptomes of skeletal muscles from WT and ZBED6-deficient pigs during sepsis. (**A**) Schematic illustration of the core promoter structure of *DOCK3* with putative ZBED6 binding site (ZBS). TSS represents the transcription start site in *DOCK3* promoter. (**B**) Cell-based reporter assays were performed in pig primary satellite cells of ZBED6-deficient and WT pigs transfected with the indicated DOCK3-ZBS-LUC reporter plasmids. Data are expressed as mean ± SEM; *$p < 0.05$, **$p < 0.01$, ***$p < 0.001$.

Table S1. Patients' characteristics.

Patients' characteristics	Patients enrolled	Controls
**Number**	25	15
**Age [years]**	64.2 (42/74)	64 (43/76)
**Gender [male/female (%)]**	13/12 (52/48)	8/7 (53.33/46.67)
**BMI [kg/m^2]**	22.3 (17.3/25.5)	ND
**MRC-sumscore**	41.4(37/47)	60
**Diagnosis [n (%)]**	Sepsis: n = 25 (100)	NA
**Survivors [n (%)]**	25(100)	15 (100)
**Severity of illness (at ICU admission)**	ICU scores:<br>SOFA: 8 (6/10)<br>APACHE-II: 24.5 (21/28)	NA

BMI, body mass index; ICU, intensive care unit; MRC, Medical Research Council; NA, not applicable; ND, not determined; SOFA, Sequential Organ Failure Assessment score; APACHE-II, Acute Physiology and Chronic Health Evaluation II.

Table S2. The potential genes bound by ZBED6 in their proximal promoters. (Excel)

Table S3. Primers and shRNAs used in this study.

**DOCK3 promoter-reporter primer**	
**1 region cloning for pGL3-basic**	
1 For	5'-CGGGGTACCATCGCCTCTCGCGGATTTC-3'
1 Rev	5'-CCCAAGCTTACGCCGTATTTCTCTTCCTCC-3'
**2 region cloning for pGL3-basic**	
2 For	5'-CGGGGTACC CGGGCGTCTTGCGAG-3'
2 Rev	5'-CCCAAGCTTACGCCGTATTTCTCTTCCTCC-3'
**3 region cloning for pGL3-basic**	
3 For	5'-CGGGGTACC CCTGGTACTAAGACGCCTGC-3'
3 Rev	5'-CCCAAGCTTACGCCGTATTTCTCTTCCTCC-3'
**ChIP-qPCR primers**	
For	5'-CGGGGTACCATCGCCTCTCGCGGATTTC-3'
Rev	5'-CCCAAGCTTACTCCGTTCCTCACAGCTTAG-3'
**qPCR primers**	
human-ZBED6 For	5'-AGCTTCCAATGACCCTGAGC-3'
human-ZBED6 Rev	5'-CAGGTGTACTGGGGGTCAAC-3'
human-FBXO30 For	5'-ACAAATGGAGACTGTGTGGCATC-3'
human-FBXO30 Rev	5'-GCCATTAGGCAAAGCACTGGATG-3'
human-FBXO32 For	5'-TGAGCGACCTCAGCAGTTAC-3'
human-FBXO32 Rev	5'-AAGGCAGGCCGGACCAC-3'
human-GAPDH For	5'-GGAGCGAGATCCCTCCAAAAT-3'
human-GAPDH Rev	5'-GGCTGTTGTCATACTTCTCATGG-3'
pig-ZBED6 For	5'-CTGTTATGTGCTTCCCCCAC-3'
pig-ZBED6 Rev	5'-AGGGTAACTATCACACTAAGCTG-3'
pig-FBXO30 For	5'-ACTCGCTGGACAGGGTGATA-3'
pig-FBXO30 Rev	5'-ACTGCCATGGGTCTAACAAAAT-3'
pig-FBXO32 For	5'-CTGTCCATCAGTCCGTCG-3'
pig-FBXO32 Rev	5'-TCGCTATCAGTTCCAACAGC-3'
pig-GAPDH For	5'-CGGAGTGAACGGATTTGGC-3'
pig-GAPDH Rev	5'-ATGACAAGCTTCCCGTTCTC-3'
pig-TRIM63 For	5'-GAGATGTTTACCAAGCCGGT-3'
pig-TRIM63 Rev	5'-CTGGTCCAGTAGGGATTTGC-3'
pig-UBB For	5'-GCATTGTTGGCGGTTTCG-3'
pig-UBB Rev	5'-CGAAGATCTGCATTTTGACCTG-3'
pig-CTSL For	5'-TGCAAATACAAGCCCCAGAG-3'
pig-CTSL Rev	5'-ATGCCCCAAGTATTACCCCA-3'
pig-SOX18 For	5'-CGAGTTCGACCAGTACCTCA-3'
pig-SOX18 Rev	5'-GGCGCTTCAAAACTTAACCC-3'
pig-DOCK3 For	5'-GGGTGTGATATCCGCAATGA-3'
pig-DOCK3 Rev	5'-CTCCCCAGCGAGGACTATTA-3'
pig-C14orf39 For	5'-TCCCATCACATCAGAGAAGAGA-3'
pig-C14orf39 Rev	5'-ATACCCAAGAGCTAAGCACC-3'
pig-G0S2 For	5'-GGAGCCGAGATGGAAACGAT-3'
pig-G0S2 Rev	5'-CAAGTCCAAACGGAAGGCGA-3'
**ZBED6 and DOCK3 shRNAs**	
shNC	GATCCGTTCTCCGAACGTGTCACGTAATTCAAGAGATTACGTG ACACGTTCGGAGAATTTTTTC
ZBED6 shRNA1	GATCCGCCTTAATTCCTGGAACAAGACTCGAGTCTTGTTCCAG GAATTAAGGCTTTTTTG
ZBED6 shRNA2	GATCCGGACCTTACTGACTCTGATTCCTCGAGGAATCAGAGTC AGTAAGGTCCTTTTTTG
ZBED6 shRNA3	GATCCGCAGAGCAGGACACTCTTATGCTCGAGCATAAGAGTG TCCTGCTCTGCTTTTTTG
DOCK3 shRNA1	GATCCGCCTGCACTACCGAAGACCATATGGACTCGAGTCCAT ATGGTCTTCGGTAGTGCAGGTTTTTTG
DOCK3 shRNA2	GATCCGCAGCAGAACTGATTCGGCAAGACCACTCGAGTGGTC TTGCCGAATCAGTTCTGCTGTTTTTTG
DOCK3 shRNA3	GATCCGCGCTACCAGGAGGCCTTCTTTGATACTCGAGTATCAA AGAAGGCCTCCTGGTAGCGTTTTTTG

Table S4. Antibody information used in this study.

Antibodies	Source	Identifier
ZBED6	ATLAS	HPA068807
Tubulin	Proteintech	11224-1-AP
FOXO3A	Immunoway	YT1763
p-FOXO3A	Immunoway	YP0115
ATROGIN-1	Immunoway	YN3108
Ubiquitin	Immunoway	YT5498
Anti-Puromycin	Sigma	MABE343
DOCK3	Proteintech	20683-1-AP
p-AKT Ser473	CST	# 4060
AKT	CST	# 9272
